# Hepatocyte Thorns, A Novel Drug-Induced Stress Response in Human and Mouse Liver Spheroids

**DOI:** 10.3390/cells11101597

**Published:** 2022-05-10

**Authors:** Chris S. Pridgeon, Dian P. Bolhuis, Filip Milosavljević, Marina Manojlović, Ákos Végvári, Massimiliano Gaetani, Marin M. Jukić, Magnus Ingelman-Sundberg

**Affiliations:** 1 Department of Physiology and Pharmacology, Karolinska Institutet, 171 65 Stockholm, Sweden; christopher.pridgeon@ki.se (C.S.P.); dian.bolhuis@gmail.com (D.P.B.); marin.jukic@ki.se (M.M.J.); 2 Faculty of Pharmacy, University of Belgrade, 11221 Belgrade, Serbia; filip.milosavljevic@pharmacy.bg.ac.rs (F.M.); marina.manojlovic@pharmacy.bg.ac.rs (M.M.); 3 Proteomics Biomedicum, Division of Physiological Chemistry I, Department of Medical Biochemistry and Biophysics, Karolinska Institute, 171 77 Stockholm, Sweden; akos.vegvari@ki.se; 4 Chemical Proteomics, Division of Physiological Chemistry I, Department of Medical Biochemistry and Biophysics, Karolinska Institute, 171 77 Stockholm, Sweden; massimiliano.gaetani@ki.se; 5 Unit of Chemical Proteomics, Science for Life Laboratory (SciLifeLab), 171 65 Stockholm, Sweden

**Keywords:** hepatocytes, spheroids, thorns, hepatotoxicity, keratins, 3D culture

## Abstract

The in vivo-relevant phenotype of 3D liver spheroids allows for long-term studies of, e.g., novel mechanisms of chronic drug-induced liver toxicity. Using this system, we present a novel drug-induced stress response in human and murine hepatocyte spheroids, wherein long slender filaments form after chronic treatment with four different drugs, of which three are PPARα antagonists. The morphology of the thorns varies between donors and the compounds used. They are mainly composed of diverse protein fibres, which are glycosylated. Their formation is inhibited by treatment with fatty acids or antioxidants. Treatment of mice with GW6471 revealed changes in gene and protein expression, such as those in the spheroids. In addition, similar changes in keratin expression were seen following the treatment of hepatotoxic drugs, including aflatoxin B1, paracetamol, chlorpromazine, cyclosporine, and ketoconazole. We suggest that thorn formation may be indicative of hepatocyte metaplasia in response to toxicity and that more focus should be placed on alterations of ECM-derived protein expression as biomarkers of liver disease and chronic drug-induced hepatotoxicity, changes that can be studied in stable in vivo-like hepatic cell systems, such as the spheroids.

## 1. Introduction

Liver spheroids from primary human hepatocytes (PHH) offer a unique combination of longevity and metabolic relevance [1] that makes them ideal for modelling chronic and acute hepatotoxicity and complex toxicities, such as extracellular matrix (ECM) stress (e.g., fibrosis) in vitro. Models, such as cancer cell lines and stem-cell-derived models, are phenotypically very different from in vivo hepatocytes and thus lack metabolic relevance. Furthermore, 2D PHH cultures dedifferentiate rapidly after removal from the donor’s blood supply and are therefore unsuitable for studies that investigate long-term phenotypes, such as chronic toxicity [2]. Furthermore, drug-induced liver toxicity in vivo includes events, such as alteration of cell proliferation, cell de- or trans-differentiation, senescence, apoptosis, delayed mitochondrial toxicity and many other mechanisms that are challenging to study in iPSC-derived or 2D hepatocyte cultures. In contrast, PHH spheroids are stable for up to 5 weeks and maintain a phenotype that is comparable to human liver tissue [1].

Modelling the complex toxicity phenomena found in the liver in vitro is difficult; therefore, toxicity is often summarised by the effects of treatment on ATP levels as an indicator of cell death. Whilst useful in routine screening, this approach can be overly reductive and miss the nuanced spectrum of stress responses that can occur before, or as an alternative to, cell death. Stress effects involving the ECM or cytoskeleton can occur in response to toxicity; for example, ethanol can activate hepatic stellate cells (HSCs), causing excessive ECM deposition and fibrosis [3]; menadione has been reported to cause cytoskeletal abnormalities in rat hepatocytes [4]; amiodarone can cause hepatic fibrosis after chronic treatment [5]. In addition, drug treatments can induce the formation of reactive oxygen or nitrogen species (ROS/RNS), either directly or through their metabolism by enzymes, such as CYP2E1 [6]. These ROS and RNS can subsequently activate HSCs, leading to similar deposition of excess ECM. For example, Mallory–Denk bodies, a type of hyaline inclusion observed in hepatocytes containing misfolded intermediate filaments including cytokeratins (CKs), can form in response to drugs, including griseofulvin, and are observed in chronic ethanol toxicity [7].

The in vivo-relevant phenotype and stability of PHH spheroids [1] allow the study of novel mechanisms for chronic hepatic drug toxicity that had previously not been possible in 2D cultures, including analyses of long-term drug-induced toxicity and ECM stress. In this study, we present a novel drug-induced stress response in PHH and primary mouse hepatocyte (PMH) spheroids wherein long slender filaments, which we have termed thorns, form after treatment with four different compounds (GW6471, MK886, NXT629, and BAY41-2272) with apparently distinct targets.

## 2. Materials and Methods

### 2.1. Cell Culture

Cryopreserved PHHs from 3 donors were obtained from 2 commercial sources (BioIVT, West Sussex, UK; and KalyCell, Plobsheim, France, Supplementary Table S1). C57/BL6 and CD1 mouse hepatocytes were obtained from Lonza (Basel, Switzerland) and BioIVT, respectively (Supplementary Table S1). Hepatocytes were thawed using INVITROGRO CP medium with TORPEDO antibiotic or INVITROGRO CP rodent medium with rodent TORPEDO, as appropriate (all BioIVT), and diluted in William’s E medium (Thermo Fisher Scientific, Waltham, MA, USA) supplemented with L-glutamine (Sigma Aldrich, St. Louis, MO, USA), insulin, transferrin, and selenium (Thermo Fisher Scientific), dexamethasone (Sigma Aldrich) and 10% (*v*/*v*) FBS (Thermo Fisher Scientific) (seeding medium). A total of 1500 cells were seeded per well of a 96-well ultra-low attachment plate (Corning, Corning, NY, USA; Thermo Fisher Scientific; faCellitate, Mannheim, Germany) in a volume of 100 µL and centrifuged (180× *g*, 150 s) before incubation under standard tissue culture conditions (37 °C, 5% CO_2_) for 5 days. On day 5, 50 µL of the seeding medium was changed with William’s E medium supplemented with L-glutamine, insulin, transferrin, selenium, and dexamethasone (culture medium).

All drug treatments were initiated 7 days after seeding to allow the spheroid formation to occur, after which 90 µL of the medium was removed and replaced with a culture medium with 0.1% (*v*/*v*) DMSO containing the drug of interest or as a vehicle control. Media containing either drug or vehicle was changed every 72 h thereafter. Treatment with free fatty acids (FFAs) was performed as described previously [8]. Briefly, a 1:1 ratio of palmitic and oleic acid was solubilised in ethanol and conjugated to 10% (*v*/*v*) FBS. Spheroids were treated with a final concentration of 480 µM FFA or vehicle added to the culture medium to a total of 3% (*v*/*v*) on day 7 after seeding.

Thorn formation was induced with one of four different compounds, GW6471 (Sigma Aldrich), MK886 (Tocris, Bristol, UK), NXT629 (MedChemExpress, Monmouth Junction, NJ, USA), or BAY41-2272 (MedChemExpress) (Supplementary Table S2). All compounds were dissolved in DMSO to a final concentration of 0.1% (*v*/*v*). Drug treatments were typically for 72 h except in the case of MK886, which often produced thorns in less than 24 h.

### 2.2. ATP Assay

Viability was assessed by quantification of cellular ATP using the CellTiter-Glo^®^ Luminescent Cell Viability Assay (Promega, Madison, WI, USA) adapting the manufacturer’s protocol. Briefly, 70 µL of the medium was removed from the spheroids and 30 µL of the reagent was added. The mixture was incubated at 37 °C for 20 min before reading using a MicroBeta^2^ LumiJET Microplate Counter (PerkinElmer, Waltham, MA, USA).

### 2.3. Thorn Isolation

To isolate the thorns from the spheroids, their brittle nature was exploited. Spheroids with thorns were pooled in 1× PBS (−) (Thermo Fisher Scientific) and the thorns were detached from the spheroid body by trituration. Intact spheroid bodies were briefly allowed to sediment under gravity and the supernatant was collected. The supernatant was centrifuged (17,000× *g*, 30 min, 4 °C) and approximately 80% was discarded. The remaining supernatant was pooled and centrifugation was repeated. Enrichment for thorns was confirmed by microscopic examination of the enriched suspension.

### 2.4. Deglycosylation and Glycosylation Staining

Deglycosylation was performed using the Protein Deglycosylation Mix II kit (New England Biolabs, Ipswich, MA, USA) following the manufacturer’s protocol. Glycosylation staining was performed using the Pierce Glycoprotein Staining kit (Thermo Fisher Scientific) according to the manufacturer’s protocol. Deglycosylation was also arbitrarily applied to whole spheroids with thorns; in these cases, the protein lysate in the manufacturer’s protocol was substituted for a spheroid suspension and deglycosylation was allowed to proceed for 4 h.

### 2.5. Whole Spheroid Staining

Spheroids were fixed in 4% (*v*/*v*) paraformaldehyde (Sigma Aldrich) overnight at 4 °C before washing three times in 1× PBS (−). Spheroids were blocked in PBTA (5% BSA, 0.25% Triton X-100 and 0.1% NaN_3_ in PBS (−)) for 2 h before incubation with the primary antibody (antibody dilutions are shown in Supplementary Table S3) at 4 °C overnight. Spheroids were then washed three times in PBS (−) before incubation with the secondary antibody at 4 °C overnight. Spheroids were subsequently washed in PBS (−) containing Hoechst 33,342 (0.1% (*v*/*v*), Thermo Fisher Scientific) and washed a further two times in PBS (−).

Whole spheroid imaging was performed in clear-bottomed plates using an LSM710, LSM700, LSM800, or LSM880 confocal microscope (Carl Zeiss, Oberkochen, Germany). Aperture settings were set to one airy unit and z-stacks were performed with either 2.5 µM or 5 µM sections. Exposure limits were established with secondary-only controls, where the primary antibody incubation was replaced with incubation with PBTA.

For quantification, maximum intensity projections of z-stacks were produced using Zen Black (Carl Zeiss). Images were then quantified using ImageJ. The channel of interest was quantified using the measure function of ImageJ, where only a single spheroid was included per image, and image size was kept constant.

### 2.6. RT-qPCR

Spheroids were lysed in Qiazol (Qiagen, Hilden, Germany) and a standard phenol-chloroform extraction was performed. Reverse transcription was performed using SuperScript III reverse transcriptase (Thermo Fisher Scientific). TaqMan chemistry and a 7500 Fast Real-Time PCR system (Thermo Fisher Scientific) were used for RT-qPCR analyses.

### 2.7. Proteomics

#### 2.7.1. Qualitative Proteomics

Two spheroid samples (50 µM GW6471 72h and vehicle control) were washed in PBS (−) and lysed in 15% (*v*/*v*) 8M urea, 100 mM NaCl, and 50 mM (NH_4_)HCO_3_ buffer (AmBic, Sigma Aldrich). Next, 15% (*v*/*v*) 1% ProteaseMax (Promega), 10% acetonitrile in AmBic was added, and pH was adjusted to 8.5 with AmBic. Samples were sonicated using a bath and probe, then freeze-thawed in liquid nitrogen three times before centrifugation (18,000× *g*, 10 min, 4 °C). The protein content of the supernatant was determined by bicinchoninic acid (BCA) assay; 10 µg protein was reduced with 500 mM dithiothreitol (DTT) in AmBic to a final concentration of 7 mM (45 min, 37 °C). Samples were alkylated with 500 mM iodoacetamide in AmBic to a final concentration of 18 mM (room temperature, 30 min), followed by digestion with 0.5 µg of trypsin (overnight, 37 °C). Samples were acidified with a final concentration of 5% formic acid, and HyperSep C-18 plates were used for clean-up (5–7 µL bed volume) before drying in a SpeedVac (Eppendorf, Hamburg, Germany). LC-MS/MS was performed by a 4/12 µL injection of 3 µg of protein, with a 90-min gradient acquired on a Q-Exactive HF mass spectrometer (Thermo Fisher Scientific). Following the identification of proteins in Mascot Server v2.5.1 using the human database, applying a 5% FDR cut-off, approximately 20% of the identified proteins were unique for each sample.

#### 2.7.2. Quantitative Proteomics

Triplicate samples, treated with either 100 µM MK886 or 0.1% (*v*/*v*) DMSO control for 72 h, and were washed in PBS (−) and lysed in 10 µL of 8 M urea, 50 mM Tris-HCl, with pH 8.5 and 8 µL of 1% ProteaseMax, 10% 50 mM acetonitrile, 100 mM Tris-HCl, and 62 µL of 100 mM Tris-HCl, pH 8.5 (including protease inhibitors). Samples were sonicated using a bath and probe before centrifugation (18,000× *g*, 10 min, 4 °C). The protein content of the supernatant was determined by BCA assay; 25 µg protein from each sample was reduced with 100 mM DTT in AmBic to a final concentration of 7 mM (37 °C, 45 min) and alkylated with 100 mM chloroacetamide in AmBic to a final concentration of 17 mM (room temperature, 30 min). Samples were digested with 0.5 µg of trypsin (overnight, 37 °C) and acidified with a final concentration of 5% formic acid. HyperSep C-18 plates were used for clean-up (5–7 µL bed volume) before drying in a SpeedVac. Samples were prepared for TMT6 labelling, 24.5 µg of each sample was dissolved in 50 mM triethylammonium bicarbonate and combined with 100 µg of TMT label in dry acetonitrile and centrifuged (550 rpm, 2 h, room temperature). The reaction was stopped with 5% hydroxylamine to a final concentration of 0.5%, centrifuged (550 rpm, 15 min, room temperature), and combined in a single tube. The labelled sample was dried in a SpeedVac, and 22 µg was cleaned with a C-18 HyperSep plate (40 µL bed volume) and StageTips before drying with a SpeedVac. The resolubilised peptides were analysed on a Q-Exactive HF mass spectrometer using a 90-min linear gradient elution on a 50 cm long C18 column, acquiring HCD MS/MS data. The TKO-TMT standard was used for QC injection (500 ng).

Protein identification was performed using the human database and detected proteins were filtered by applying a 1% FDR cut-off using a Percolator. Proteome Discoverer v2.5 (Thermo Fisher Scientific) was used to determine the relative protein abundances in the sample groups using a TMT quantification method. Quantification was provided based on reporter ion intensities and protein abundance ratios were calculated using median values for each treatment group. The Student’s *t*-test was applied as a hypothesis test.

#### 2.7.3. Proteome Integral Solubility Alteration (PISA) Analysis

Proteome Integral Solubility Alteration (PISA) analysis exploits the drug-dependent changes in protein solubility. This is assessed by quantitative proteomic analyses of the soluble fraction after the treatment of the spheroids with drugs or vehicles and incubation of equal portions of each sample at different temperatures. Protein interactions of these drugs can then increase or decrease the soluble amount of the protein in the whole range of temperature tested, which is confidently measured by proteomics using a statistically robust number (>3) of biological replicates.

The PISA assay was carried out in a 16-plex format, using the PISA assay method as published [9] and adapted to the TMTpro 16-plex technology [10]. Briefly, PISA thermal treatment was performed at 16 temperatures (44–60 °C), and extraction was performed in PISA, with the addition of a 0.4% NP40 incubation step for 30 min at 4 °C after five freeze/thaw cycles, then, the soluble part was separated by ultracentrifugation and collected separately from the pellet.

The total protein content of the sample was determined by a micro-BCA assay. Fifty μg of each sample was processed as follows: reduction with 8 mM dithiothreitol (55 °C, 45 min), alkylation with 25 mM iodoacetamide (30 min, 25 °C, in darkness), protein precipitation overnight at −20 °C using sample:cold acetone in a ratio of 1:5. After precipitation, the supernatant was discarded and protein pellets were resuspended in 20 mM EPPS containing 8 M urea, then samples were progressively diluted in 20 mM EPPS to a lower urea concentration and digested using LysC enzyme at 30 °C for 6h at the substrate:enzyme ratio of 1:75, followed by trypsin digestion overnight at 37 °C (enzyme:substrate ratio of 1:50). The digested proteins were labelled using TMTpro 6-plex for two hours and pooled in the final 16-plex sample according to the manufacturer’s protocol. The 16-plex sample was cleaned and desalted using the C18 SepPack column (Waters, Milford, MA, USA) and fractionated into 48 fractions using reverse phase C18 at a capillary flow and high pH. Each fraction was dried by SpeedVac, resolubilized, and injected into a nanoLC-ESI-MS, eluted at 300 nL/min over a 90-min linear gradient on a heated 50 cm long C18 column, and data were acquired by a Q-Exactive HF mass spectrometer with HCD fragmentation and MS/MS analysis. The identification and quantification of the peptides and proteins from the RAW data of all fractions were obtained by the MaxQuant database search engine against the complete Uniprot human database, using a 1% FDR cut-off. Quantification was based on the reporter ion intensities after the normalisation of each total TMT label intensity across the whole dataset, with only 2 or more unique peptides per protein included in the final PISA analysis. The ratios of soluble protein abundance in PISA and Student’s *t*-test were measured in normalised values over the untreated controls.

### 2.8. Scanning Electron Microscopy

Specimens were fixed by immersion in 2.5% glutaraldehyde in 0.1 M phosphate buffer, pH 7.4. Following fixation, specimens were adhered to poly-lysine pre-treated Nunc™ Thermanox™ coverslips (Thermo Fisher Scientific), washed with 0.1 M phosphate buffer, pH 7.4, followed by MilliQ water. The coverslips were then subjected to stepwise ethanol dehydration, transferred to acetone, and critical-point-dried using carbon dioxide (Leica EM CPD030). The coverslips were finally mounted on specimen stubs using carbon adhesive tabs and sputter-coated with a 10 nm layer of platinum (Quorum Q150T ES). SEM images were acquired using an Ultra 55 field emission scanning electron microscope (Zeiss) at 3 kV and the SE2 detector.

### 2.9. Animal Experiments

Animal experiments were conducted at the University of Belgrade, Faculty of Pharmacy, in accordance with the ethical guidelines of the University of Belgrade. Ethical permission was granted by The Ministry of Agriculture, Forestry and Water Management, Veterinary Directorate of the Republic of Serbia (number 119-01-4/11/2020-09). Forty-eight adult female Swiss Webster mice were obtained from the Medical Military Academy animal farm and housed in a 12-h dark/light cycle in a temperature and humidity controlled, specific pathogen-free environment, with ad libitum access to pelleted food and water. Mice were acclimatised for fourteen days upon arrival, after which they were divided into 3 groups of 16 animals and injected intraperitoneally with either 5 mg/kg/day GW6471 for 14 days, vehicle control (10 mL/kg/day) for 14 days or vehicle control for 7 days followed by GW6471 for 7 days. To prepare for the treatment, 5 mg of GW6471 was dissolved in 1 mL of DMSO and the obtained DMSO solution was mixed with 9 mL distilled water; the DMSO and distilled water mix (1:9) was used as a vehicle. Animals were sacrificed by cervical dislocation and the livers were harvested. Half of each liver was snap-frozen in liquid nitrogen and a half was fixed in 4% formaldehyde overnight and embedded for cryosectioning. Tissues were shipped to the Karolinska Institutet; an export permit was granted by The Ministry of Health of the Republic of Serbia (515-07-09073/2021-07).

### 2.10. Masson’s Trichrome Staining

Eight µm cryosections were taken from fixed OCT-embedded liver tissue and stained using the Masson’s Trichrome Stain Kit (Polysciences, Warrington, PA, USA) following the manufacturer’s protocol. Slides were imaged using a Zeiss Axioplan brightfield microscope (Carl Zeiss) at 20× and 40× magnification.

## 3. Results

### 3.1. Chronic Drug Treatment Causes the Appearance of Thorn-Like Structures in Spheroid 3D Liver Model

**Figure 1:**
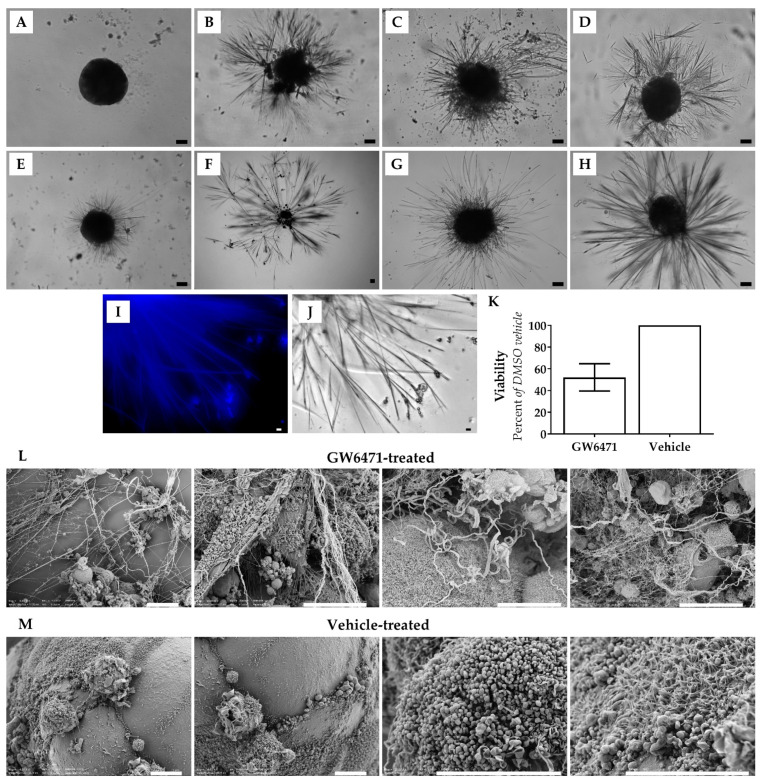
Examples of Thorn Morphology. Brightfield micrographs showing: (**A**) PHH spheroid exhibiting typical morphology. (**B**) Donor 1 PHH spheroid treated with 100 µM MK886 for 12 h. (**C**) Donor 1 PHH treated with 50 µM GW6471 for 72 h. (**D**) Donor 2 PHH spheroid treated with 7.8 µM NXT629 for 168 h. (**E**) Donor 2 PHH spheroid treated with 10 µM BAY41-2272 for 72 h. (**F**) Donor 1 PHH spheroids treated with 10 µM MK886 for 10 days (**G**) C57BL/6 PMH spheroid treated with 50 µM GW6471 for 96 h. (**H**) CD1 PMH hepatocytes treated with 100 µM MK886 for 120 h. (**I**) Blue autofluorescence of thorns in the GW6471-treated donor 1 spheroid. (**J**) Thorns in a GW6471-treated donor 1 spheroid, shown in panel I. (**K**) Viability of GW6471-treated spheroids at 72 h relative to vehicle control, error bars are SEM. Scanning electron micrographs of donor 1 spheroids with: (**L**) 50 µM GW6471 for 72 h and (**M**) vehicle treatment (0.1% (*v*/*v*) DMSO) for 72 h. Scale bars are 10 µm.

Thorn formation was first observed after treatment of human liver spheroids with 10 µM GW6471 for 14 days to investigate the effects of PPARα antagonism (Figure 1). PHH and PMH spheroids were subsequently treated with two other PPARα antagonists, MK886 and NXT629, and the soluble guanylate cyclase (sGC) activator BAY41-2272, all of which also induced thorn formation (Figure 1). Thorns were observed protruding from the spheroid body after different durations of drug treatment (1–14 days), depending on the compound and concentration (Figure 1B–E). Thorns were often longer than the spheroid body diameter (i.e., >200 µm) and exhibited a branching structure (Figure 1). The thorns were rigid, brittle, insoluble in aqueous solution or 90% (*v*/*v*) DMSO, and adhered to the ultra-low attachment plates. In addition, thorns exhibited blue autofluorescence (Figure 1I). By increasing the drug concentration of GW6471 from 10 µM to 50 µM, the time for thorns to form was reduced from 10 days to 72 h (Figure 1B). In the case of MK886, thorns formed within 10 days (Figure 1F) when treated with 10 µM and within 12 h when the dose was increased to 100 µM (Figure 1B). Thorn formation, using NXT629 and BAY41-2272, was observed at 72 h of treatment and was not observed in all spheroids. Spheroids treated with thorn-forming compounds exhibited decreased ATP content and after 72 h of treatment with GW6471, the ATP was reduced by 50% (Figure 1K). DMSO control spheroids retained ATP content and were smooth and rounded without large projections (Figure 1A). Scanning electron microscopy (SEM) revealed the altered morphology of GW6471-treated spheroids in detail; thorns were abundant and protruding from the cell surface and appeared to be composed of multiple thinner filaments (Figure 1L). In contrast, vehicle-treated spheroids showed a smoother surface without large projections or disruptions, though they show a ciliated-like appearance in some areas (Figure 1M). In summary, three PPARα antagonists and an sCG activator induced the formation of characteristic thorn-like structures in the PHH spheroid model. The extent of the thorn formation was proportional to the drug concentration and incubation time.

### 3.2. Thorns Are Composed of Glycosylated Protein Fibres

**Figure 2:**
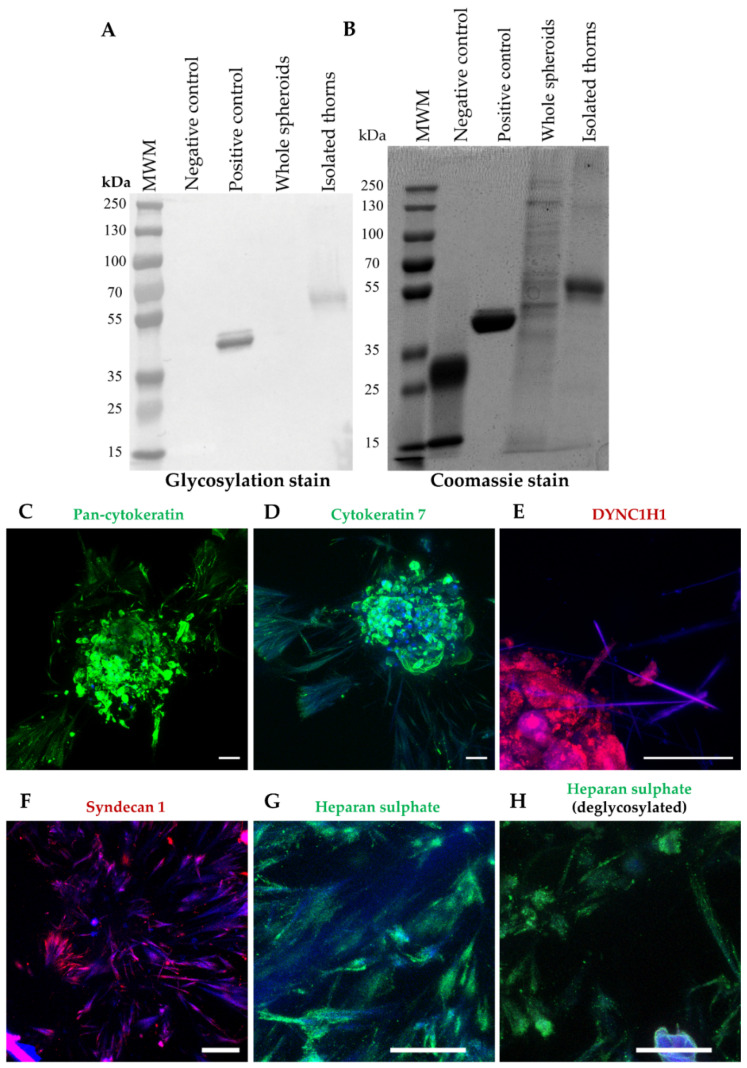
Thorn composition. Glycosylation and Coomassie staining of thorns isolated from donor 1 PHH spheroids treated with 50 µM GW64712 for 72 h. (**A**) Glycosylation stain. (**B**) Coomassie stain. Immunofluorescence staining of thorns on donor 1 spheroids. (**C**) pan-CK with deglycosylation, (**D**) CK 7, (**E**) cytoplasmic dynein heavy chain 1, (**F**) syndecan 1, (**G**) heparan sulphate without deglycosylation, (**H**) heparan sulphate with deglycosylation. Scale bars are 50 µm.

The morphology of the thorns suggests that they are the result of the stress-induced formation of ECM proteins, which are often glycosylated [11]. The degree of glycosylation of isolated thorn fractions was examined. Thorns were produced from spheroids treated with 50 µM GW6471 for 72 h and isolated by trituration and centrifugation to separate the thorn structures. The enrichment of the thorns was confirmed by microscopic inspection. Glycosylation staining revealed that the thorn preparation had a high amount of glycosylated protein, around 55–70 kDa, which was not apparent in similar analyses of the whole spheroid lysate (Figure 2A). The band stained for glycosylation was equivalent to a band of similar weight in the Coomassie stain (Figure 2B). These results indicate enrichment of glycosylated proteins in the thorns.

To determine the exact composition of the thorns, proteomic analyses were conducted. Initially, a qualitative screen of the PHH spheroids treated with 50 µM GW6471 or 0.1% (*v*/*v*) DMSO for 72 h was performed, in which 1269 proteins were detected (data available upon request). Next, triplicate samples of the PHH spheroids treated with 100 µM MK886 or 0.1% (*v*/*v*) DMSO for 72 h were analysed. The initial extraction left an insoluble fraction that was larger in the MK886-treated spheroids and, therefore, was investigated for thorn proteins using a more stringent dissolution method.

Two independent analyses were performed with these samples: an analysis of the insoluble fraction in isolation and then combined with the soluble fraction in a label-free analysis. These analyses detected 2676 and 2737 proteins, respectively. The proteins with the highest total spectrum counts, which were at least 10-fold increased above the DMSO treatment in the insoluble pellet, and the combined analysis, are shown in Table 1 and Table 2, respectively. Considerable homology between the experiments was observed, with 22 proteins similarly upregulated (Table 3). Ten of these proteins were associated with the cytoskeleton, of which eight were cellular filaments, e.g., CKs and tubulins. Interestingly, CK7 and cytoplasmic dynein 1 heavy chain 1 (DYNC1H1) were increased by MK886 treatment, which was later confirmed by immunofluorescence staining (Figure 2). Based on the proteomic analyses (Table 1 and Table 2, further data available upon request) a panel of candidate thorn proteins was selected for examination with immunohistochemistry. Spheroids were treated with GW6471 for 72 h, with MK886 for 24 h, or with 0.1% (*v*/*v*) DMSO vehicle control, and the whole spheroids were examined by immunohistochemistry. Positive staining was obtained for pan-CK, syndecan 1, DYNC1H1, CK7, and heparan sulphate, either with or without prior deglycosylation (Figure 2G,H). Staining for CK7, syndecan 1, and heparan sulphate were seen, regardless of deglycosylation, whereas pan-CK stained positively only after deglycosylation (Figure 2). The thorns remained positive for heparan sulphate after deglycosylation, but the intensity of staining was decreased (Figure 2G,H). Treatment with GW6471 or MK886 increased the expression of CK7 (Supplementary Figure S1E). Pan-CK staining was validated by Western blotting on an enriched mouse thorn preparation compared with whole spheroids, demonstrating an enrichment of the CK expression in the thorns (Supplementary Figure S2). In summary, the thorns are composed of various glycosylated cytoskeleton-associated proteins.

### 3.3. Thorns Form Due to Lipid Metabolism Disturbances and Their Creation Is Stimulated by Reactive Oxygen Species

**Figure 3:**
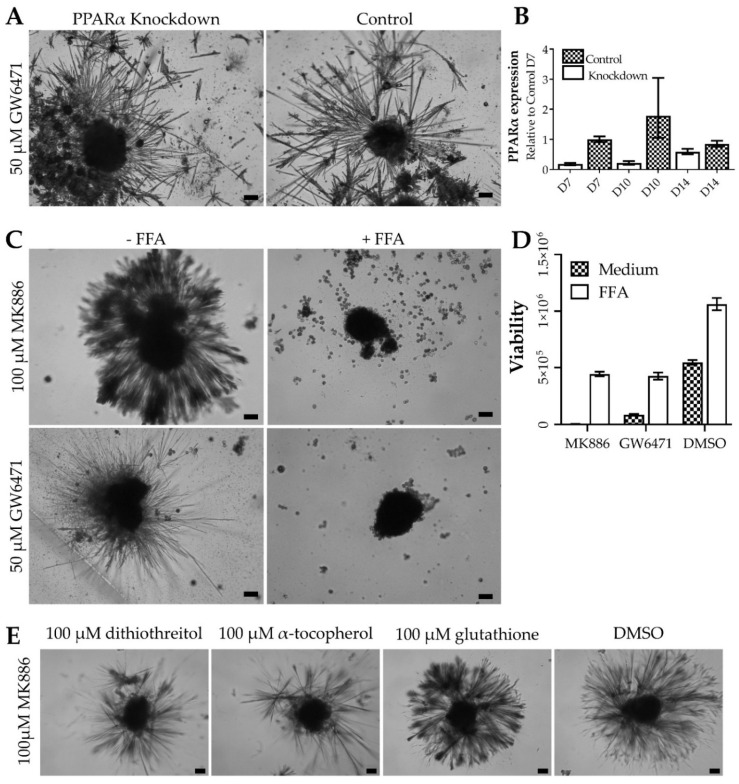
Mechanisms of thorn formation. (**A**) Brightfield micrographs showing the effects of siRNA-mediated PPARα knockdown thorn formation in donor 1 PHH spheroids. Spheroids were treated with 50 µM GW6471 for 72 h. (**B**) PPARα knockdown validated by qPCR of PPARα. (**C**) Brightfield micrographs of thorn formation in donor 1 PHH spheroids after treatment with 50 µM GW6471 or 100 µM MK886 with or without FFA supplementation for 72 h. (**D**) Viability of donor 1 spheroids treated, as described in panel C. (**E**). Brightfield micrographs of donor 1 PHH spheroids treated with 100 µM MK886 with either 100 µM α-tocopherol, 100 µM dithiothreitol, 100 µM glutathione, or 0.1% (*v*/*v*) DMSO. Scale bars are 10 µm.

The role of PPARα in thorn formation was investigated by siRNA-mediated knockdown of PPARα in PHH spheroids. Analyses of PPARα silenced spheroids revealed a reduction in PPARα mRNA expression and reduced CYP3A4 expression, which is transcriptionally regulated by PPARα [12], demonstrating efficient silencing of PPARα expression (Supplementary Figure S1). However, knockdown of PPARα did not induce thorn formation alone or affect thorn formation upon treatment with GW6471 for 72 h (Figure 3). This indicates an off-target action of GW6471, independent of PPARα antagonism. In addition, the modulation of PPARδ and PPARγ with GW0742 and T0070907, respectively, did not elicit spindle formation, either alone or in combination (data not shown).

Reactive oxygen species are often involved in cytotoxicity, therefore their role in the formation of thorns was evaluated by treatment with different antioxidants. Either α-tocopherol, glutathione, dithiothreitol (at concentrations reported in the literature [13,14,15]), or vehicle was added to the spheroids treated with MK886 or GW6471. Dithiothreitol and α-tocopherol reduced the extent of the thorn formation caused by both MK886 and GW6471 treatments (Figure 3, Supplementary Figure S1). Interestingly, in the presence of GW6471, α-tocopherol caused a round halo of extra-spheroidal matrix, distinct in shape from the thorns, giving rise to the halo of material observed in Supplementary Figure S1.

To evaluate the possible common targets for the PPARα antagonists causing the thorn formation, common proteins affected by the binding of MK886 and GW6471 were examined in an unbiased manner using PISA analysis [9]. PISA analysis aims to identify proteins that interact with the treatment compound, which is based on the change in their solubility with thermal treatment. Spheroids were treated for two hours with GW6471, MK886, or a 0.1% (*v*/*v*) DMSO vehicle control before subjection to PISA analysis.

**Figure 4:**
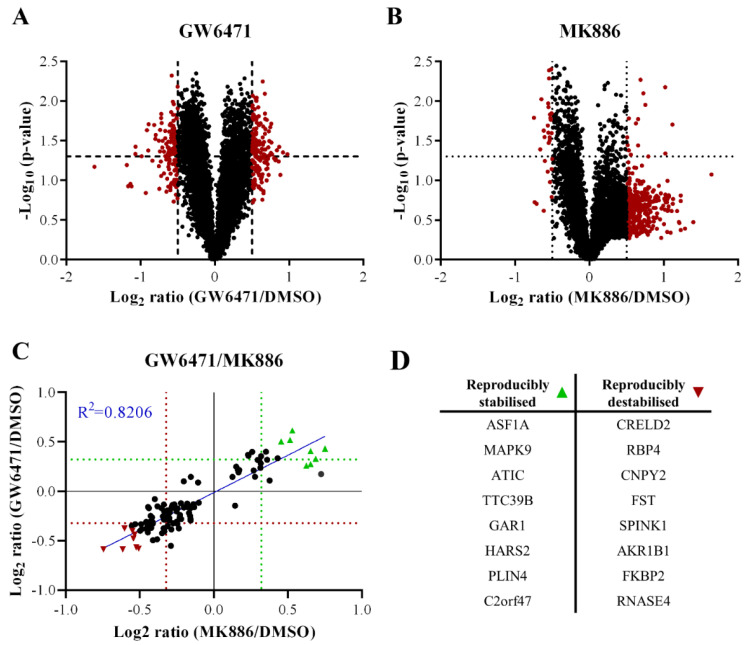
PISA analysis of donor 1 PHH spheroids treated with 50 µM GW6471, 100 µM MK886 or 0.1% (*v*/*v*) DMSO for 2 h. In all panels, the dotted lines indicate a threshold of 0.4 change in protein amount ratio or a p-value threshold of 0.05. (**A**) Volcano plot showing the fold change in soluble amount after thermal treatment (Log_2_ ratio) and statistical significance expressed as −Log_10_ (*p*-value) of GW6471 and DMSO-treated spheroids. (**B**) Volcano plot showing the fold change in soluble amount after thermal treatment (Log_2_ ratio) and statistical significance expressed as −Log_10_ (*p*-value) of MK886 and DMSO-treated spheroids versus DMSO. (**C**) Correlation between similarly altered proteins in GW6471 and MK886 treatments. The proteins significantly increased in soluble amounts in both GW6471 and MK886 treatments as shown in green triangles, while proteins labelled with red triangles are significantly and commonly decreased in soluble amounts. (**D**) Summary table of the significantly and commonly altered proteins labelled in panel C.

Compared to DMSO, GW6471, treatment stabilised 501 proteins and destabilised 805, whereas MK886 treatment stabilised 80 proteins and destabilised 252. In some cases, the protein stability was affected similarly by both compounds (Figure 4). A total of 108 proteins had reproducibly altered stabilisation by both treatments, of which 77 were destabilised and 31 were stabilised (data available upon request), indicating somewhat similar properties for both compounds. At least 16 of the 108 reproducibly altered proteins have been reported to interact with lipid metabolism, 17 to interact with neurite outgrowth or endothelial sprouting, and 16 to interact with the regulation of the ECM (data available upon request).

Since PPARα, -δ, and -γ are involved in the regulation of lipid metabolism [16,17], it was hypothesised that the modulation of these receptors could cause metabolic stress, including inhibition of lipid synthesis. Thus, the effects of free fatty acids (FFA) on thorn formation by MK886 and GW6471 were studied (Figure 3, Supplementary Figure S1). The addition of 480 µM palmitic and oleic acid in a 1:1 ratio, which approximates the concentration in serum [8], abolished thorn formation upon MK886 or GW6471 treatment for 72 h (Figure 3). In addition, FFA supplementation prevented much of the decrease in ATP caused by drug treatment (Figure 3D).

In summary, thorn formation appears to constitute a stress response, which also included the formation of reactive oxygen species induced by the alteration of the lipid homeostasis within the spheroids—reactions that can be ameliorated by FFA supplementation and antioxidants. Further experiments are required for a more detailed identification of the underlying mechanisms.

### 3.4. Thorns Represent Chronic Drug Treatment-Induced Hepatocyte Stress Response

Initial proteomics analyses suggested several CKs as candidates for the thorn component proteins (Table 1 and Table 2), and immunofluorescence demonstrated positive staining in the thorns for pan-CK and CK 7 (Figure 2). Since CK18 is commonly found in hepatocytes, it was investigated if CK7 and CK18 were essential for thorn formation. Both proteins were subjected to siRNA-mediated knockdown, however, despite effective knockdown of gene expression, assessed by qPCR, thorn formation after treatment with 50 µM GW6471 for 72 h was not prevented (data not shown).

Since thorn formation occurred in response to several drugs and the thorns were pan-cytokeratin and CK7 positive, it was considered that similar keratin expression might occur in response to treatment with non-thorn-forming compounds. PHH spheroids were treated with aflatoxin B1 (200 nM), paracetamol (3.75 mM), chlorpromazine (5 µM), cyclosporine A (10 µM), ketoconazole (30 µM), or vehicle control for 72 h. Gene expression of CK7, CK8, CK18, and CK19 was examined by qPCR, and CK7 and CK18 by immunofluorescence (Supplementary Figure S3A,B). Aflatoxin B1, paracetamol, and ketoconazole increased protein expression of CK7 and CK18 (Supplementary Figure S3A). CK7 mRNA expression was not induced by any treatment, but CK18 mRNA was induced by aflatoxin B1, chlorpromazine, and paracetamol (Supplementary Figure S3B). These data indicate that both CK7 and CK18 may constitute biomarkers for drug-induced stress in PHH spheroids.

### 3.5. Chronically GW6471-Treated Mice Exhibit Fibre Accumulation in the Liver

**Figure 5:**
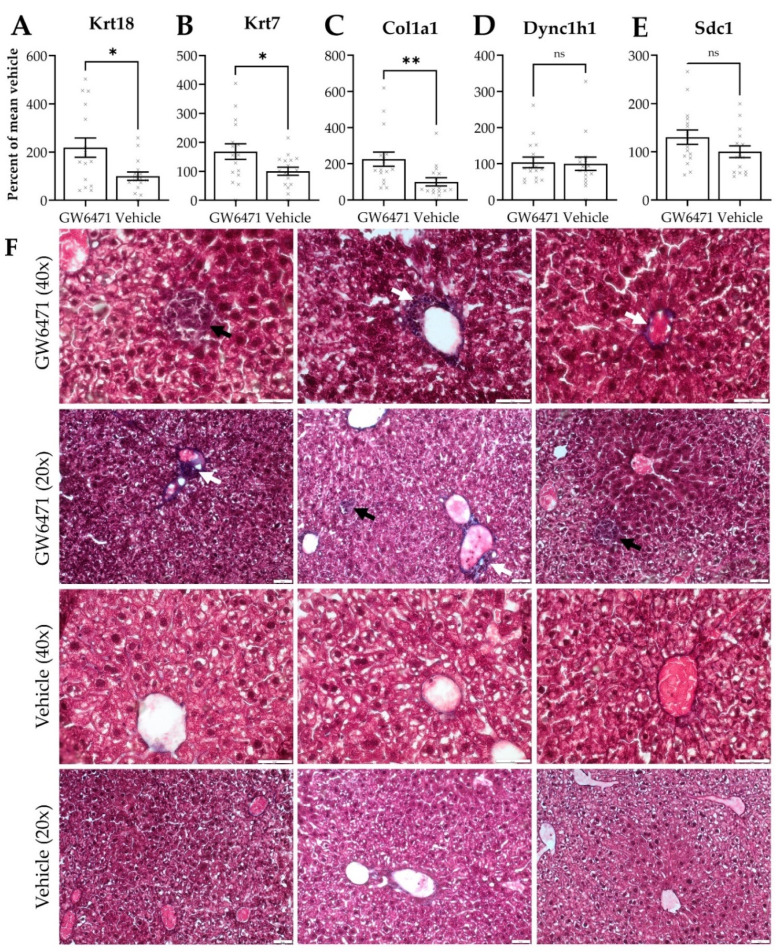

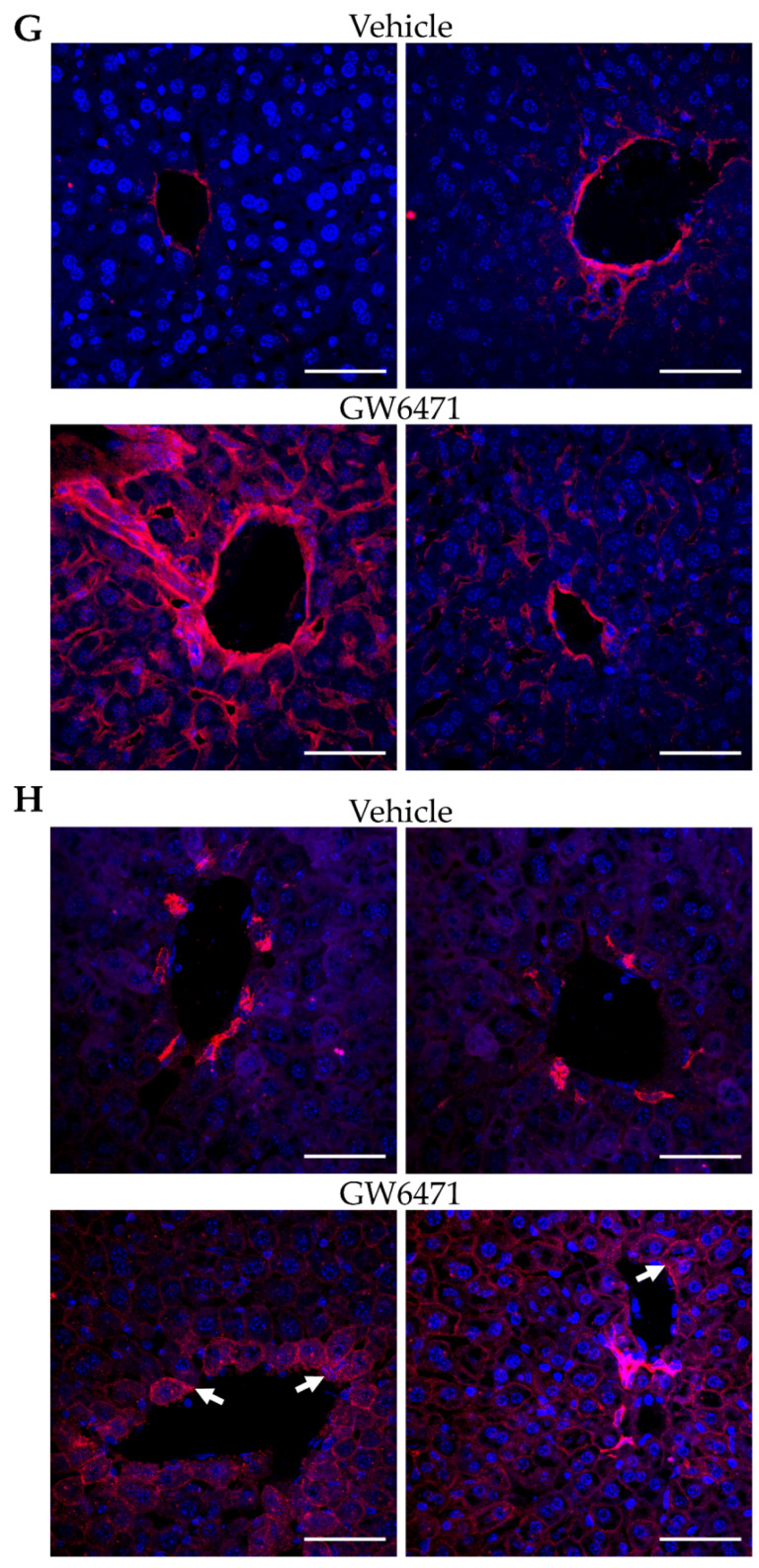
Gene expression and histological staining in mouse liver treated with GW6471—fold change in gene expression of (**A**) Krt18, (**B**) Krt7, (**C**) Col1a1, (**D**) Dync1h1 and (**E**) Sdc1 in liver tissue of mice treated with GW6471 (5 mg/kg/day) or vehicle (10 mL/kg/day 10% (*v*/*v*) DMSO in distilled water) for two weeks. Each cross shows the expression in a single animal. Bars show the mean, error bars are SEM, percentages calculated using 2^−ΔΔCT^ and are expressed relative to the mean of 1, Tbp was the control gene. Asterisks denote significance as determined by an unpaired two-tailed Student’s *t*-test * = *p* ≤ 0.05, ** = *p* ≤ 0.01. (**F**) Masson’s Trichrome staining of mouse livers treated with vehicle or 50 µM GW6471 for two weeks. Collagen visualised in purple/black. Black arrowheads show foci of collagen enriched cells. White arrowheads show increased collagen deposition around blood vessels. 20× and 40× magnification. Immunofluorescence of (**G**) collagen 1 and (**H**) pan-cytokeratin expression in mouse tissue sections treated with vehicle or with 50 µM GW6471 for two weeks. White arrowheads indicate the accumulation of keratin staining around the blood vessels in GW6471-treated mice. The left and right images show two separate fields from the same condition. All sections are 8 µm. Scale bars are 50 µm.

Female Swiss Webster mice were treated with GW6471 and the effects on hepatic thorn protein gene expression were investigated by qPCR. Expression of Krt7, Krt18, and Col1a1 was significantly increased in animals treated with GW6471 over vehicle-treated controls (Figure 5A–E). Masson’s Trichrome staining of the same tissues revealed a tendency for increased collagen deposition around the blood vessels in animals treated with GW6471 compared to vehicle controls (Figure 5F, white arrows). In addition, foci of darkly staining cells, indicating collagen deposition, were observed in GW6471-treated animals but not in those treated with vehicle controls (Figure 5F, black arrows). The increased expression of collagen was confirmed by immunofluorescence staining in liver sections, showing increased collagen 1 staining around the blood vessels after treatment with GW6471 for two weeks (Figure 5G); increased pan-CK staining was also observed surrounding the hepatic blood vessels after GW6471 treatment (Figure 5H, white arrows). In summary, the capability of GW6471 to increase thorn protein deposition in the liver was also translated to an in vivo system, as it was apparent from collagen and cytokeratin accumulation in GW6471-treated mouse livers.

## 4. Discussion

### 4.1. Thorn Characteristics

PHH spheroids offer an in vitro liver model that has improved stability and phenotype compared to the 2D cultures [1]. Their utility and in vivo-like phenotype have been demonstrated [18] and make them suitable for the study of novel chronic toxicity responses. This report focuses on a novel phenomenon where PHH spheroids produce long, slender projections from the spheroid body, visually reminiscent of thorns, in response to chronic treatment with several drugs. These thorns consist of glycosylated protein fibres, which exhibit heterogeneous protein composition and represent an easily detectable readout of chronic drug-induced stress in spheroids. Their formation apparently occurs due to disturbances in lipid metabolism and is facilitated by oxidative stress, mediated by reactive oxygen species, as evident from the inhibition of thorn formation following treatment with antioxidants. The thorns morphologically resemble neurite outgrowth in sensory ganglia exposed to nerve growth factor [19] and endothelial sprouting [20], but are morphologically and mechanistically distinct. Thorns exhibit blue autofluorescence, which may offer insight into their composition. Collagen, keratin, NAPDH, arachidonic acid, and bile salts exhibit blue autofluorescence, all of which are present in the liver [21,22,23]. The presence of keratins in vitro and collagen in vivo indicate that these are the likely sources of the autofluorescence observed.

Chronic treatment of mice with GW6471 induces accumulation of ECM protein fibres, including cytokeratin 7 and collagen in mice. A similar accumulation of cytokeratin 7 in thorns indicates that thorn formation mimics a chronic drug-induced stress response that occurs in vivo. Although collagen was increased in vivo in mice, it was not observed in the thorns and is most likely due to the absence of Stellate cells in the PHH spheroids that are known to be the major source for collagen production after differentiation to myofibroblasts [24].

Initially, thorn formation was observed serendipitously after treatment with the PPARα antagonist, GW6471, whilst investigating the effects of PPARα modulation in PHH spheroids. Thorn formation was later observed after treatment with MK886 and NXT629, which are both also PPARα antagonists and MK886 is also an inhibitor of leukotriene biosynthesis [25]. Thorn formation with BAY41-2272, a nitric oxide-independent activator of soluble guanylate cyclase [26], was also observed by chance. Of these drugs, only MK886 has been used in clinical trials, for the treatment of asthma and psoriasis, with only moderate success [27,28]. The precise morphology of the thorns was variable and dependent on the drug used (Figure 1). MK886 appeared to produce denser and more branching thorn structures compared with GW6471, which produced more slender individual thorns. The thorns appeared to become longer with extended treatment, e.g., 10 days of treatment with MK886 produced longer thorns than after 12 h with 100 µM of MK886. The cause of the altered morphology is unclear, however, it may be related to the propensity for each compound to induce thorn formation.

### 4.2. Proposed Mechanisms of Thorn Formation

Proteomic and immunohistochemical analyses revealed many constituent proteins, including cytokeratins, spectrins, filamins, talin, syndecan 1, and tubulins induced during thorn formation. Using immunohistochemistry, cytoplasmic dynein heavy chain 1 (DYNC1H1), syndecan 1, CK7, and heparan sulphate were identified in the thorns and were also present in the spheroid body (Figure 2). Three of the thorn-forming compounds are PPARα antagonists; however, effective siRNA-mediated knockdown of PPARα did not influence the extent of GW6471-induced thorn formation (Figure 3). In addition, modulation of PPARδ or PPARγ with T0070907 and GW0742, respectively, had no effect, indicating off-target effects of the PPARα antagonists (data not shown). PISA analysis was used to identify common targets of GW6471 and MK886, and proteins affecting lipid metabolism were highly enriched among the set of targeted proteins. Subsequent experiments revealed that the addition of FFA or the antioxidants abrogated thorn formation (Figure 3). It can therefore be postulated that the PPARα antagonists affect lipid homeostasis in PHH spheroids, causing oxidative stress and increased synthesis of several proteins, leading to thorn formation.

CK7 expression was increased in response to the treatment with PPARα antagonists and was observed in the thorns (Supplementary Figure S1E). However, typically, CK7 is only expressed in cholangiocytes and hepatic progenitor cells, not in mature hepatocytes [29]. Under periods of stress, including cholestatic diseases [29], prolonged alcohol use [30], or hepatocellular neoplasms, hepatocytes can undergo metaplasia and express CK7, which has been described as an escape mechanism for toxicity in hepatocytes [31]. Therefore, it is possible that thorn formation is an example of hepatocyte metaplasia, where excess CK7 and other proteins form the thorns due to drug-induced ECM stress.

A similar result of drug-induced ECM stress is seen during the formation of Mallory–Denk bodies in response to chronic ethanol toxicity or by treatment with the antifungal agent griseofulvin [7]. Mallory–Denk bodies are a type of hyaline inclusion observed in hepatocytes and are characterised by the presence of misfolded intermediate filaments, including cytokeratins. Thus, it is likely that the drug-induced cellular stress results in different morphological changes (thorns or Mallory–Denk bodies) in ECM expression related to the type of stress that is induced.

The suitability of CK7 as a biomarker of drug-induced hepatotoxic effects was investigated, and it was found that both aflatoxin B1, paracetamol, and ketoconazole induced its expression. In addition, CK18, previously described as a hepatocyte stress marker, was induced by aflatoxin B1, chlorpromazine, and paracetamol. These findings underscore the importance of the examination of the different kinds of cytokeratins as markers for drug-induced hepatocyte stress and toxicity.

### 4.3. Comparable Effects of GW6471 in Mice

The expression of thorn proteins, following the treatment with GW6471 in mice, was monitored. GW6471-treated mice showed increased hepatic expression of Krt7, Krt18, and Col1a1 (Figure 5), which is consistent with in vitro findings. Additionally, Masson’s Trichrome and immunofluorescence staining showed increased collagen and keratin deposition around the hepatic blood vessels in GW6471 treatment, suggesting that thorn formation manifests as a deposition of CKs and collagen in vivo.

Thorn formation represents a rapidly forming and easily detectable readout of toxicity that, in some cases, is visible even to the naked eye. Observations of thorn formation or an increase in CK7 and CK18 expression may be useful in drug screening approaches for drug-induced liver injury. PHH spheroids acquire thorn protein expression similarly to mice in vivo, but more rapidly. This positions them for rapid screening for toxicity that emerges after chronic treatment, e.g., hepatic fibrosis caused by amiodarone [5].

### 4.4. Conclusions

In conclusion, these results demonstrate a heretofore undescribed stress response in human and mouse hepatocytes. In PHH and PMH spheroids, three PPARα antagonists caused the formation of thorns, which are composed of several proteins, including CK7, DYNC1H1, and syndecan 1 and glycosylated with heparan sulphate. The formation of thorns appears to be stress-related, showing hepatocyte metaplasia in response to toxicity, and was inhibited by supplementation with FFA and antioxidants. The long-term stability of the PHH spheroids allows the monitoring of novel aspects of chronic adverse liver responses related to drug- or disease-induced toxicity. Monitoring for alterations or induction of thorn proteins, such as cytokeratins, could serve as biomarkers for the determination of pathological and drug-induced alterations of liver function.

Even though PHH spheroids represent the best currently attainable model to study chronic drug-induced liver phenotypes, it remains an artificial in vitro system, which in fact, represents an accumulation of hepatocytes resistant to dedifferentiation. In contrast, the human liver is a complex multicellular system containing non-parenchymal cells, which are perfused with blood and all of its contents. Therefore, in vivo human hepatocytes are normally exposed to circulating free fatty acids and antioxidants, which were used in several experiments herein and impacted upon thorn formation. Consequently, thorns likely represent an over-propagated response to a situation that would occur in the human liver in vivo after chronic drug treatment. However, translation into an in vivo system indicates that the accumulation of protein fibres in mice chronically treated with GW6471 is an equivalent phenomenon to the thorn formation in spheroids chronically treated with the same drug; translation into an in vivo human system is unfortunately not possible. Many more proteins were enriched in proteomics analysis than were detected by immunofluorescence. This may be in part due to the structure and glycosylation of the thorns, which may sterically hinder antibody binding.

## Figures and Tables

**Table 1:** Proteomics analysis of ECM fraction of donor 1 PHH spheroids treated with 100 µM MK886 for 72 h. The 31 most highly expressed proteins in MK886 treatment that are >10-fold increased by the drug versus DMSO in the ECM fraction alone. A total of 2676 proteins were analysed. Data are from triplicate MK886/DMSO ECM fractions. ‘Sum of MK886′ or ‘Sum of DMSO’ shows the total expression of a given protein in each treatment.

Identified Proteins	Sum of MK886	Sum of DMSO
Spectrin alpha chain, non-erythrocytic 1	355	17
Cytoplasmic dynein 1 heavy chain 1	331	5
Filamin-A	327	10
Spectrin beta chain, non-erythrocytic 1	319	32
CK, type I cytoskeletal 18	297	24
Filamin-B	276	7
Clathrin heavy chain 1	266	18
Plectin	261	3
Fatty acid synthase	223	9
Talin-1	213	8
DNA-dependent protein kinase catalytic subunit	207	1
Elongation factor 1-alpha 1	202	19
Tubulin beta-2B chain	201	16
Tubulin beta-4A	191	17
Ras GTPase-activating-like protein IQGAP2	186	7
Neuroblast differentiation-associated protein AHNAK	175	9
Ras GTPase-activating-like protein IQGAP1	147	1
Aldo-keto reductase family 1 member C2	144	7
eIF-2-alpha kinase activator GCN1	128	2
Complement C4-B	121	3
Elongation factor 2	118	3
Fructose-bisphosphate aldolase B	112	11
Alpha-enolase	108	8
Ribosome-binding protein 1	104	5
UTP--glucose-1-phosphate uridylyltransferase	104	3
NADPH--cytochrome P450 reductase	103	10
Cytochrome P450 2C8	101	10
Cytosolic 10-formyltetrahydrofolate dehydrogenase	101	4
Heat shock protein HSP 90-alpha	96	9
Tubulin beta chain	241	41
CK, type II cytoskeletal 7	114	16

**Table 2:** Proteomics analysis of combined soluble and ECM fraction of donor 1 PHH spheroids treated with 100 µM MK886 for 72 h. The 31 most highly expressed proteins in MK886 treatment that are >10-fold increased by MK886 versus DMSO in the combined soluble and ECM fraction. Data are from triplicate MK886/DMSO ECM fractions. ‘Sum of MK886′ or ‘Sum of DMSO’ shows the total expression of a given protein in each treatment.

Identified Proteins	Sum of MK886	Sum of DMSO
Filamin-A	480	26
CK, type I cytoskeletal 18	424	42
Cytoplasmic dynein 1 heavy chain 1	420	6
Plectin	397	3
Filamin-B	393	15
Talin-1	308	12
Fatty acid synthase	307	12
Tubulin beta-2B chain	266	16
Ras GTPase-activating-like protein IQGAP2	261	25
DNA-dependent protein kinase catalytic subunit	252	1
Elongation factor 1-alpha 1	239	24
Tubulin beta-4A chain	235	17
CK, type II cytoskeletal 7	216	18
Ras GTPase-activating-like protein IQGAP1	205	5
Aldo-keto reductase family 1 member C2	182	12
Complement C4-B	167	16
Elongation factor 2	163	6
Ribosome-binding protein 1	159	5
UTP--glucose-1-phosphate uridylyltransferase	157	10
Alpha-enolase	156	10
Coatomer subunit alpha	153	1
eIF-2-alpha kinase activator GCN1	153	5
Formimidoyltransferase-cyclodeaminase	141	13
C-1-tetrahydrofolate synthase, cytoplasmic	140	8
Gelsolin	134	9
Staphylococcal nuclease domain-containing protein 1	133	1
Cytosolic 10-formyltetrahydrofolate dehydrogenase	133	4
Aldo-keto reductase family 1 member C3	121	9
Heat shock 70 kDa protein 1A	113	6
Long-chain-fatty-acid--CoA ligase 5	112	8
Alpha-actinin-1	105	7
T-complex protein 1 subunit beta	100	2
Glutamine--fructose-6-phosphate aminotransferase 1	99	3
ATP-dependent RNA helicase A	96	1

**Table 3:** Common proteins from analyses presented in Table 1 and Table 2. Tick marks (✓) denote whether the protein is related to the cytoskeleton or is a related to or is a structural filament protein.

Common Proteins	Cytoskeleton	Filament
Cytoplasmic dynein 1 heavy chain 1	✓	✓
Filamin-A	✓	✓
CK, type I cytoskeletal 18	✓	✓
Filamin-B	✓	✓
Plectin	✓	✓
Fatty acid synthase		
Talin-1	✓	
DNA-dependent protein kinase catalytic subunit		
Elongation factor 1-alpha 1		
Tubulin beta-2B chain	✓	✓
Tubulin beta-4A	✓	✓
Ras GTPase-activating-like protein IQGAP2		
Neuroblast differentiation-associated protein AHNAK		
Ras GTPase-activating-like protein IQGAP1	✓	
Aldo-keto reductase family 1 member C2		
eIF-2-alpha kinase activator GCN1		
Complement C4-B		
Elongation factor 2		
CK, type II cytoskeletal 7	✓	✓
Alpha-enolase		
UTP--glucose-1-phosphate uridylyltransferase		
Cytosolic 10-formyltetrahydrofolate dehydrogenase		

## Data Availability

The mass spectrometry proteomics data have been deposited with the ProteomeXchange Consortium via the PRIDE [32] partner repository with the dataset identifier PXD033550.

## Supplementary Materials

The following supporting information can be downloaded at: https://www.mdpi.com/article/10.3390/cells11101597/s1, Figure S1: PPARα knockdown and antioxidant treatment, Figure S2: Pan-CK Western blot, Figure S3: CK expression in donor 1 PHH spheroids in response to toxicity; Table S1: Donor details for human and mouse hepatocytes, Table S2: Drug treatment concentrations, vehicle, and suppliers, Table S3: Antibody dilutions and suppliers.
