# Supplementary material for: Hepatocyte Thorns, A Novel Drug-Induced Stress Response in Human and Mouse Liver Spheroids
Source: Cells. 2022 May 10;11(10):1597. doi: 10.3390/cells11101597 (PMC9139950; doi:10.3390/cells11101597)
Supplement: Supplementary file 1 [file cells-11-01597-s001.zip › Supplementary Figure S1.pdf]

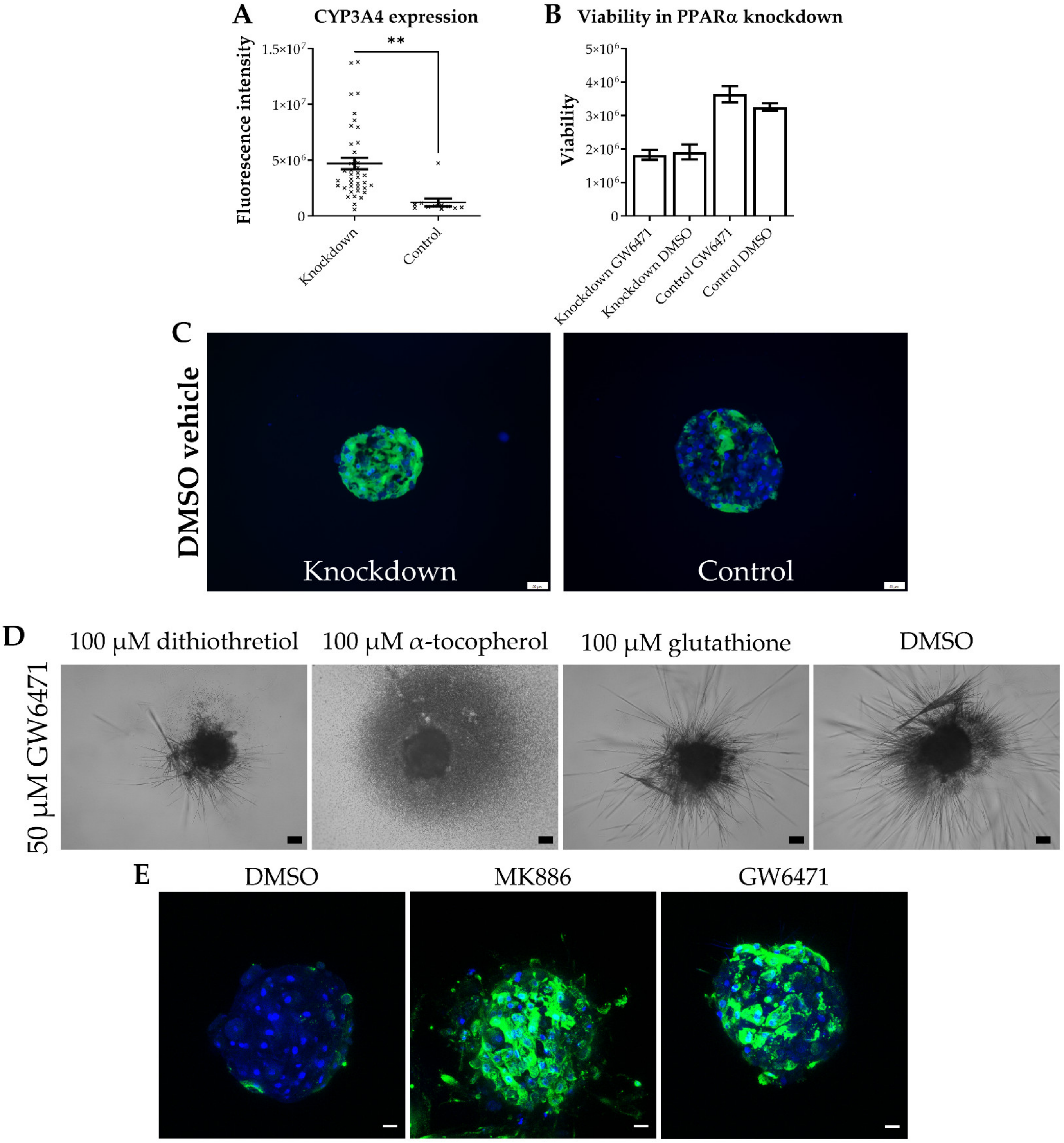

Figure S1. PPARα knockdown and antioxidant treatment. PPARα knockdown in donor 1 PHH spheroids treated with GW6471 for 72 hours. **A.** Quantitation of the knockdown presented in panel C; each point represents a single region of interest. Error bars are SEM. **B.** Viability in donor 1 PHH spheroids 72 hours after spheroid formation +/- PPARα knockdown and +/- GW6471 treatment. **C.** Fluorescence micrographs showing CYP3A4 expression (green) of donor 1 PHH spheroids with PPARα knockdown or vehicle control. **D.** Brightfield micrographs of donor 1 PHH spheroids with 72 hours of treatment of 50 µM GW6471 treatment with either 100 µM α-tocopherol, 100 µM dithiothreitol, 100 µM glutathione or 0.1% (v/v) DMSO. **E.** Immunofluorescence micrographs showing cytokeratin 7 expression (green) in donor 3 PHH spheroids after 72 hours of treatment with 0.1% (v/v) DMSO, 100 µM MK886 or 50 µM GW6471. Scale bars are 20 µm.
