# Supplementary material for: Hepatocyte Thorns, A Novel Drug-Induced Stress Response in Human and Mouse Liver Spheroids
Source: Cells. 2022 May 10;11(10):1597. doi: 10.3390/cells11101597 (PMC9139950; doi:10.3390/cells11101597)
Supplement: Supplementary file 1 [file cells-11-01597-s001.zip › Supplementary Figure S3.pdf]

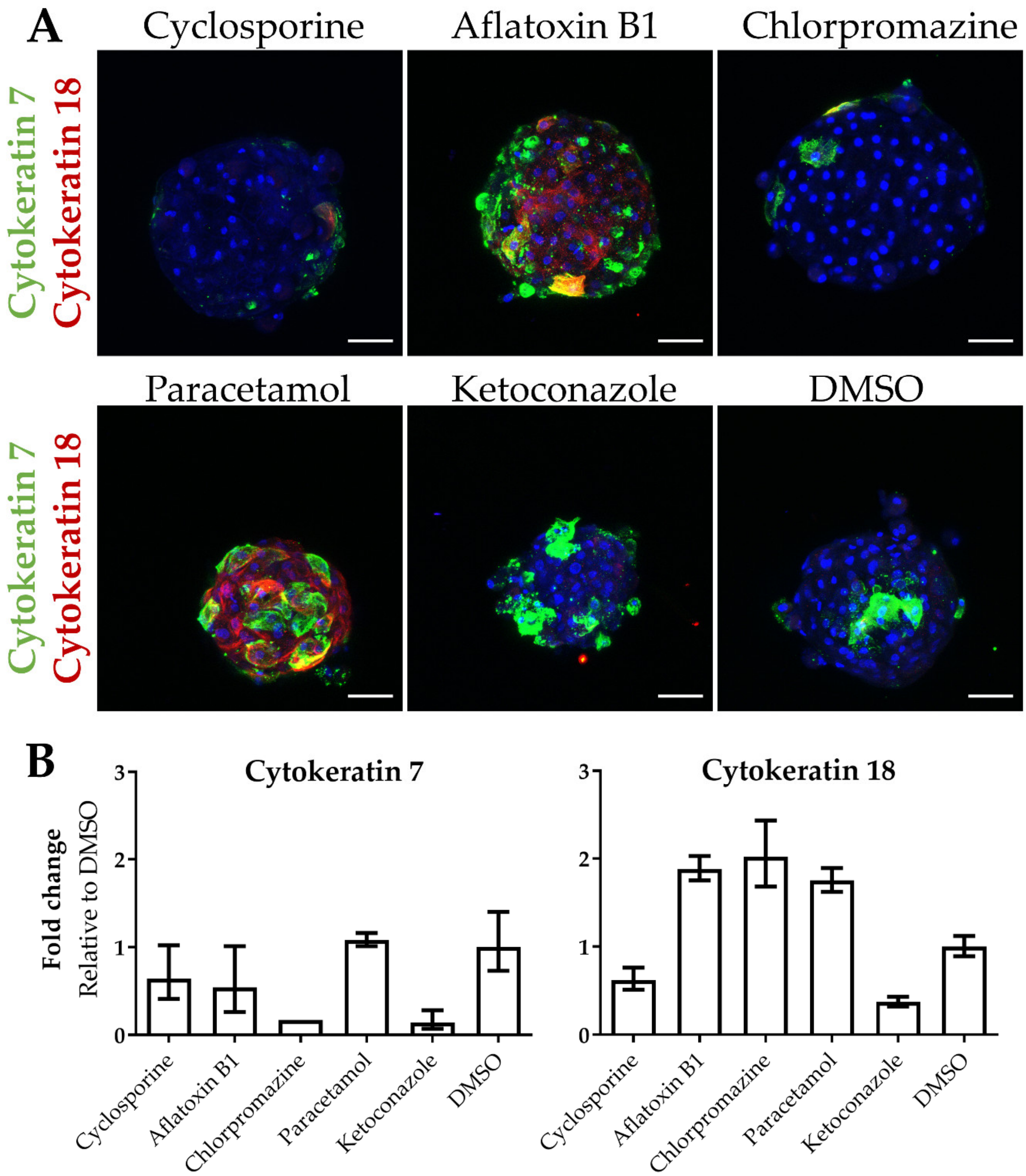

Figure S3. CK expression in donor 1 PHH spheroids in response to toxicity. **A.** Fluorescence micrographs of donor 1 spheroids showing CK 7 and 18 expression after 72 hours of treatment with either cyclosporine, aflatoxin B1, chlorpromazine, paracetamol, ketoconazole or DMSO vehicle control. **B.** Expression of CK 7 and 18 gene expression after treatment as described in panel B. Fold change values are $2^{-\Delta\Delta CT}$, error calculated as per [28]. TBP was the housekeeping gene.
