# Supplementary material for: Hepatocyte Thorns, A Novel Drug-Induced Stress Response in Human and Mouse Liver Spheroids
Source: Cells. 2022 May 10;11(10):1597. doi: 10.3390/cells11101597 (PMC9139950; doi:10.3390/cells11101597)
Supplement: Supplementary file 1 [file cells-11-01597-s001.zip › Supplementary Table S1.pdf]

Table S1. donor details for human and mouse hepatocytes.

Human Hepatocytes					
**#**	**Sex**	**Race**	**Age**	**Cause of death**	**Supplier**
Donor 1	Male	Hispanic	25	Head trauma, blunt injury	BioIVT
Donor 2	Female	Caucasian	47	Adrenocarcinoma	KalyCell
Donor 3	Female	African descent	27	Anoxia	BioIVT
Donor 4	Male	Caucasian	45	Neuroendocrine tumour	KalyCell
**Mouse Hepatocytes**					
C57/BL6	Male				Lonza
CD1	Male				BioIVT
