# Supplementary material for: Hepatocyte Thorns, A Novel Drug-Induced Stress Response in Human and Mouse Liver Spheroids
Source: Cells. 2022 May 10;11(10):1597. doi: 10.3390/cells11101597 (PMC9139950; doi:10.3390/cells11101597)
Supplement: Supplementary file 1 [file cells-11-01597-s001.zip › Supplementary Table S2.pdf]

Table S2. Drug treatment concentrations, vehicle and suppliers.

Drug name	Supplier	Target	IC50	Concentration range	Vehicle
**Spindle-forming compounds**					
GW6471	Sigma Aldrich	PPARα antagonist	240 nM	10-50 µM	0.1% (*v/v*) DMSO
BAY41-2272	MedChemExpress	Soluble guanylyl cyclase activator	-	10 µM	0.1% (*v/v*) DMSO
MK886	Tocris	5-lipoxygenase-activating protein inhibitor, PPARα antagonist	30 nM, 500-1000 nM	10-100 µM	0.1% (*v/v*) DMSO
NXT629	MedChemExpress	PPARα antagonist	77 nM	0.77-77 µM	0.1% (*v/v*) DMSO
**Other Compounds**					
TGFβ	R&D Systems	-	-	5 ng/mL	4 mM HCl, 1 mg/mL bovine serum albumin
Glutathione	Sigma Aldrich	-	-	100 µM	0.1% (v/v) DMSO
Dithiothretiol	Sigma Aldrich	-	-	100 µM	0.1% (v/v) DMSO
α-Tocopherol	Sigma Aldrich	-	-	100 µM	0.1% (v/v) DMSO
Paracetamol	Sigma Aldrich	-	-	3.75 mM	0.1% (v/v) ethanol
Ketoconazole	Sigma Aldrich	-	-	30 µM	0.1% (v/v) DMSO
Aflatoxin B1	Sigma Aldrich	-	-	200 nM	0.1% (v/v) DMSO
Cyclosporine A	Sigma Aldrich	-	-	10 µM	0.1% (v/v) DMSO
Chlopromazine	Sigma Aldrich	-	-	5 µM	0.1% (v/v) DMSO
