# Supplementary material for: Hepatocyte Thorns, A Novel Drug-Induced Stress Response in Human and Mouse Liver Spheroids
Source: Cells. 2022 May 10;11(10):1597. doi: 10.3390/cells11101597 (PMC9139950; doi:10.3390/cells11101597)
Supplement: Supplementary file 1 [file cells-11-01597-s001.zip › Supplementary Table S3.pdf]

Table S3. Antibody dilutions and suppliers.

Target	Supplier		Host	Dilution
**Primary antibodies**				
Cytokeratin 7	Abcam	AB9021	Mouse	1:100
Cytokeratin 18	Abcam	AB133263	Rabbit	1:250
CYP3A4	Nosan Corporation	PAP 011	Rabbit	1:5000
Pan-cytokeratin	Thermo Fisher Scientific	MA513203	Mouse	1:100
Pan-cytokeratin	Thermo Fisher Scientific	BS-2190R	Rabbit	1:200
Collagen 1	Novus Biologicals	NB600-408	Rabbit	1:200
Syndecan 1	Thermo Fisher Scientific	PA5-16918	Rabbit	1:100
Cytoplasmic dynein heavy chain 1	Thermo Fisher Scientific	PA5-114149	Rabbit	1:50
Heparan sulphate	AMSBio	370225-S	Mouse	1:100
**Secondary antibodies**				
AlexaFluor 488 goat anti-mouse	Thermo Fisher Scientific	A11001	Goat	1:500
AlexaFluor 555 donkey anti-rabbit	Thermo Fisher Scientific	A31572	Donkey	1:500
