# Supplementary figures and images for: Hepatocyte Thorns, A Novel Drug-Induced Stress Response in Human and Mouse Liver Spheroids
Source: Cells. 2022 May 10;11(10):1597. doi: 10.3390/cells11101597 (PMC9139950; doi:10.3390/cells11101597)

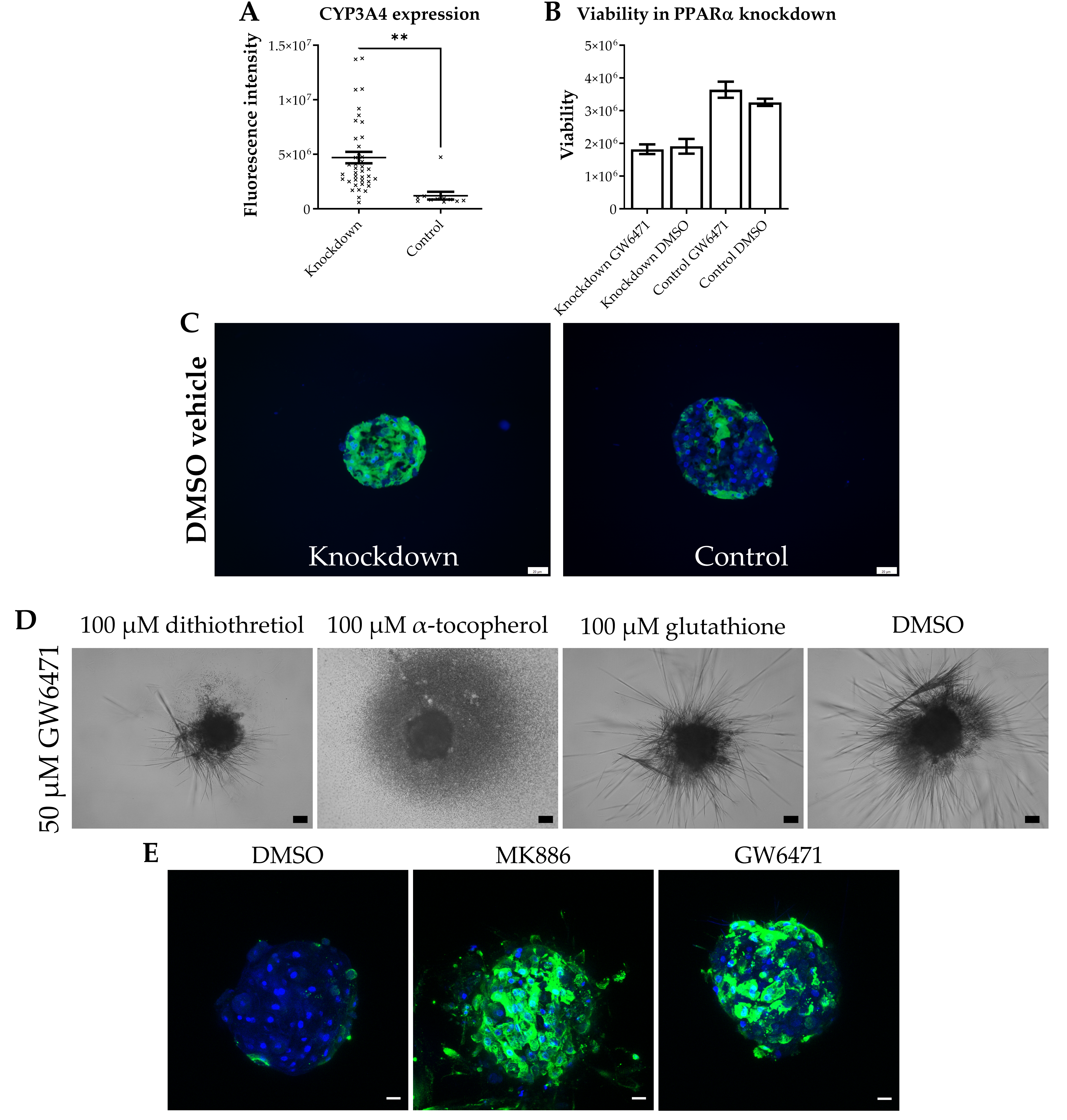

Supplement: Supplementary file 1 [file cells-11-01597-s001.zip › Supplementary Figure S1 - Mechanisms of thorn formation.tif]

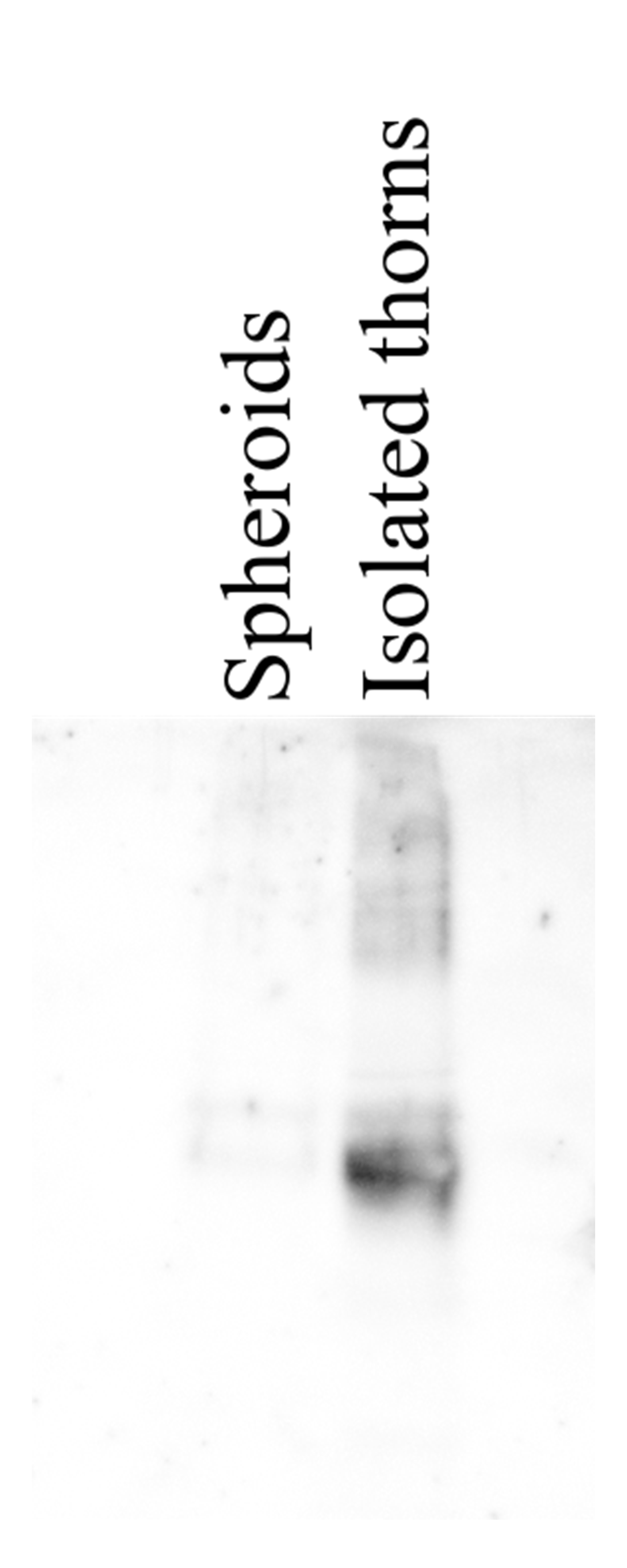

Supplement: Supplementary file 1 [file cells-11-01597-s001.zip › Supplementary Figure S2 - Pan-cytokeratin western blot.tif]

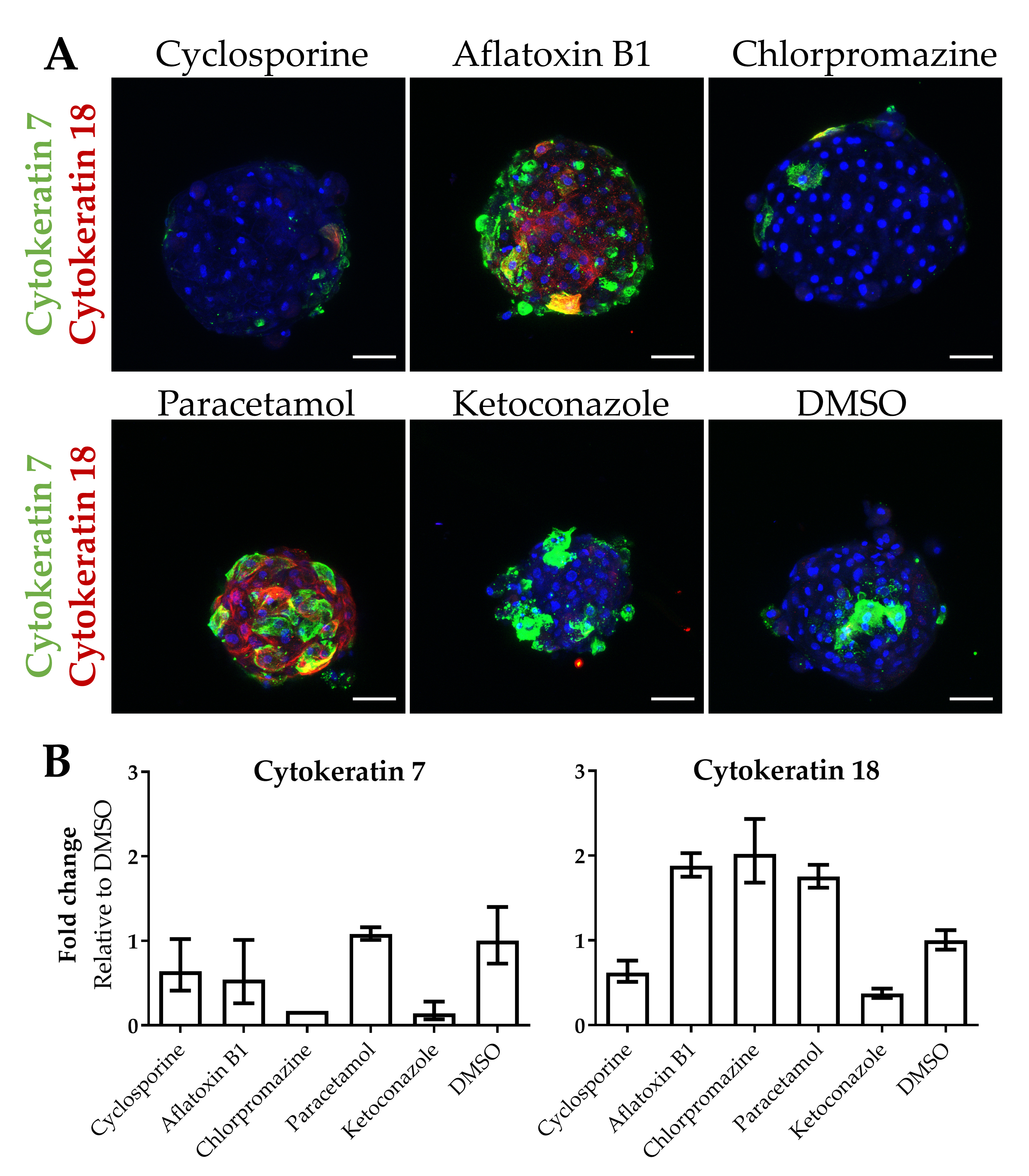

Supplement: Supplementary file 1 [file cells-11-01597-s001.zip › Supplementary Figure S3 - Keratins in thorns and toxicity.tif]
